# Supplementary figures and images for: Effect of Ankle Weights as a Frailty Prevention Strategy in the Community-Dwelling Elderly: A Preliminary Report
Source: Int J Environ Res Public Health. 2022 Jun 15;19(12):7350. doi: 10.3390/ijerph19127350 (PMC9224507; doi:10.3390/ijerph19127350)

Supplemental File  
fig. S1.

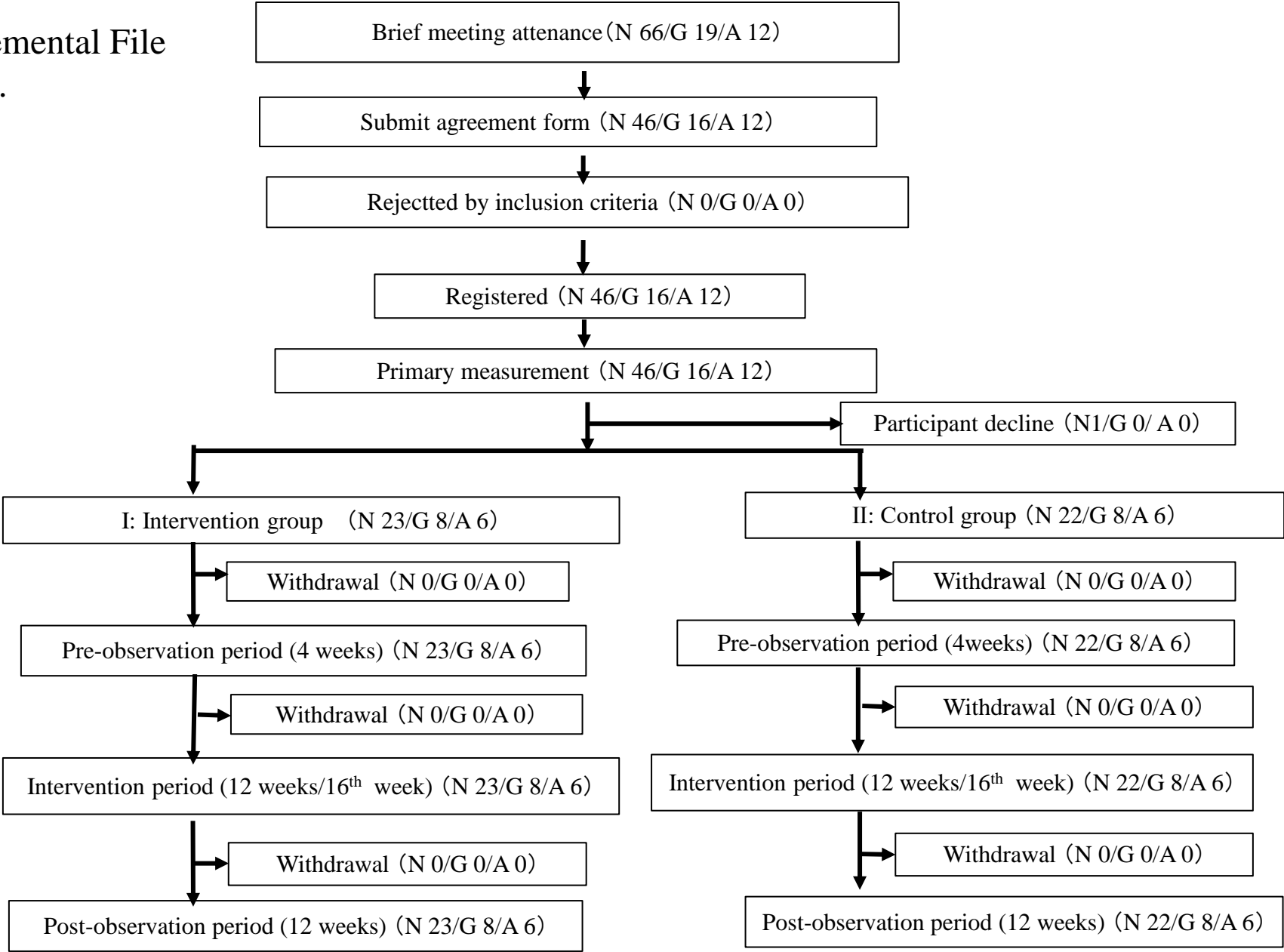

Supplement: Supplementary file 1 [file ijerph-19-07350-s001.zip › ijerph-1707705-supplementary.pdf]
